# Polarization Conversion and Optical Meron Topologies in Anisotropic Epsilon-Near-Zero Metamaterials

**DOI:** 10.1021/acsphotonics.5c00241

**Published:** 2025-05-15

**Authors:** Vittorio Aita, Anastasiia Zaleska, Henry J. Putley, Anatoly V. Zayats

**Affiliations:** † Department of Physics and London Centre for Nanotechnology, 4616 King’s College London, Strand, London WC2R 2LS, U.K.; ‡ School of Physics and Astronomy, University of Birmingham, Edgbaston, Birmingham B15 2TT, United Kingdom

**Keywords:** polarization, merons, ENZ metamaterials, spin−orbit coupling, anisotropy

## Abstract

Plasmonic metamaterials provide a flexible platform for light manipulation and polarization management thanks to their engineered optical properties with exotic dispersion regimes. Here we exploit the enhanced spin–orbit coupling induced by the strong anisotropy of plasmonic nanorod metamaterials to control the polarization of vector vortex beams and generate complex field structures with meron topology. Modifying the degree of ellipticity of the input polarization, we show how the observed meron topology can be additionally manipulated. Flexible control of the state of polarization of vortex beams is important in optical manipulation, communications, metrology, and quantum technologies.

## Graphical abstract

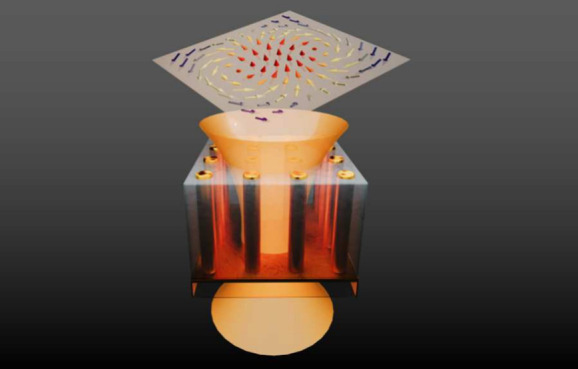

## Introduction

Polarization-controlled light–matter interactions are important in modern technologies ranging from optical communications and sensing to photochemical transformations and quantum optics.^1–7^ The ability to engineer optical beams in space and time and material properties enables precise control over their mutual influence.^8,9^ Complex topological structures, including polarization and field quasiparticles of light, were demonstrated in evanescent fields as well as propagating waves, exploiting interactions between spin and orbital angular momentum of light.^10,11^ Achieving such a high degree of control over photonic states provides an opportunity to encode high density of information with unique topological properties that can open up new opportunities in optical information processing and communications.^11–13^ Uniaxial materials are important in this respect, as they provide optical spin–orbit coupling that can be used for the generation of vortex beams.^14^ Uniaxial metamaterials can provide much stronger anisotropy than that of natural media, leading to an enhancement of both spin–orbit coupling^15^ and chiral response.^16^

Plasmonic-nanorod-based metamaterials are known for possessing various dispersion regimes arising due to their epsilon-near-zero (ENZ) properties. In the spectral range of the hyperbolic dispersion, these metamaterials exhibit strong anisotropy and respond as either a metal or a dielectric to light of different polarizations.^17^ The ENZ behavior exclusively affects fields polarized along the nanorods (parallel to the optical axis of the metamaterial) so that, under plane-wave illumination, this regime can only be accessed at oblique incidence. However, this condition on the electric field can be achieved at normal incidence by strongly focusing either scalar or vector beams to generate a non-negligible longitudinal field component.^18^ The combination of structured light with engineered plasmonic metamaterials can therefore be exploited to achieve strong spin–orbit coupling and tailor the polarization of optical fields.

The interaction of vector beams with uniaxial media can be used to control vector vortex beams and transform their polarization, for example, into azimuthal or complex vortex patterns, depending on the dispersion regime of the metamaterial.^15^ It was shown theoretically that a nonideal radially polarized beam, wherein local polarization is elliptical, develops a vorticity whose direction is mediated by the birefringence and the sign of the linear dichroism of the metamaterial through spin–orbit coupling as well as influenced by the longitudinal field in the ENZ regime.^15^ Here we experimentally demonstrate polarization control of vector vortex beams with an anisotropic plasmonic metamaterial in its ENZ and hyperbolic regimes (Figure 1). In the former case, we demonstrate the azimuthalization of the input polarization, whereas in the latter we reveal the emergence of vortex-like polarization structures that possess second-order meron topology.

**Figure 1:**
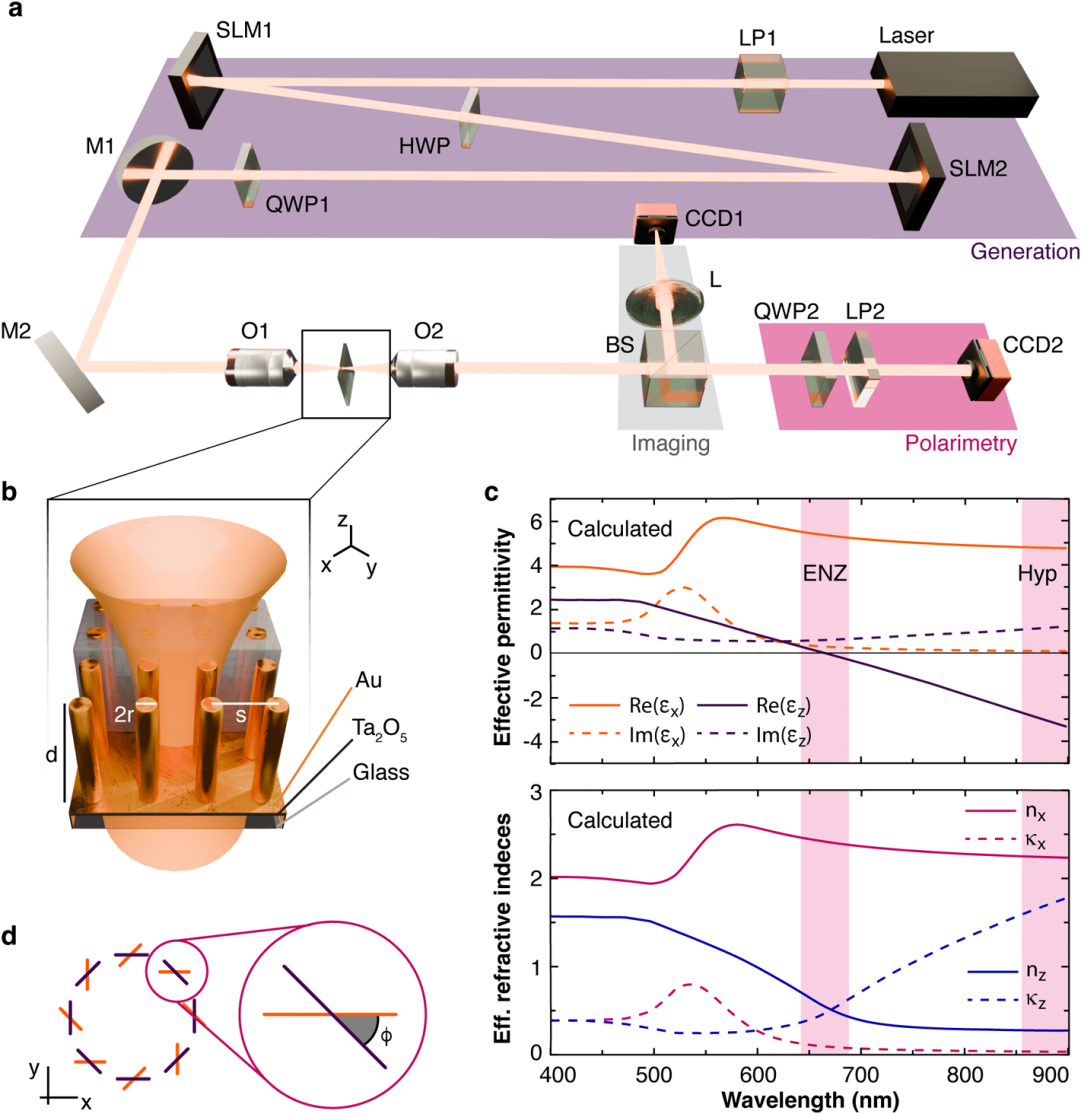
(a) Schematic of the experimental setup with (b) an illustration of the metamaterial under focused illumination. (c) Components of (top) the effective permittivity tensor and (bottom) the real (*n*) and imaginary (κ) parts of the ordinary (*x*) and extraordinary (*z*) refractive index of the metamaterial obtained with the Maxwell Garnett approximation (eq 4). (d) Local projection of an arbitrary state of polarization (orange) onto the azimuthal state (black). The inset shows the definition of the angle ϕ between them.

## Results and Discussion

Vector vortex beams are usually described as a superposition of two vortices carrying a topological charge *l* and orthogonal circular (left-handed (L) and right-handed (R)) polarization states,^19^ making it convenient to express them in terms of Laguerre–Gauss modes ${\mathrm{LG}}_{lp}$ :

1 $$|\psi \rangle =|\sigma_{1}\rangle \langle \sigma_{1}|\psi \rangle {\mathrm{LG}}_{l_{1}p}+|\sigma_{2}\rangle \langle \sigma_{2}|\psi \rangle {\mathrm{LG}}_{l_{2}p}$$

where *p* is the radial quantum number describing the LG modes, |σ_*j*_⟩ represents a circular state of polarization with spin σ_*j*_ $\left(|\sigma_{j}\rangle =(\mathrm{x̂}-i\sigma_{j}ŷ)/\sqrt{2}\right)$ , and Dirac notation is used to express the projections of the state |ψ⟩ onto the basis vectors {|R⟩, |L⟩}. By fixing the spin (σ_*i*_) and angular ( l _*i*_) momenta of each term in eq 1, the state |ψ⟩ can be obtained as a superposition of eigenmodes of the total angular momentum *J* = *L* + *S*, where *L* and *S* are the orbital and spin angular momenta, respectively. The subspace of *J* = 0 can be obtained for l = ±1 and σ = ∓1, which span the orthogonal states describing radial (|Rad⟩) and azimuthal (|Azi⟩) polarizations:

$$|Rad\rangle ={\mathrm{LG}}_{-10}\left|1\right\rangle +{\mathrm{LG}}_{10}\left|-1\right\rangle$$ 2a

2b $$|Azi\rangle ={\mathrm{LG}}_{-10}\left|1\right\rangle -{\mathrm{LG}}_{10}\left|-1\right\rangle$$

It should be noted that eq 2a does not satisfy Maxwell’s equations, and the longitudinal field of an appropriate amplitude *E_z_* should be added to ensure a divergence of zero (∇·**E** = 0),^18,20^ while the electric field of the azimuthal state (eq 2b) is perfectly two-dimensional—note that the electric displacement vector **D** should be considered in the medium.

Experimentally, we generated complex vector beams with the desired polarization structure by employing two spatial light modulators (Figure 1a,b; see Methods for the details). The beam was then focused at normal incidence along the optical axis of the metamaterial by an objective with numerical aperture NA = 0.85, and the transmitted light was collected with a second objective of NA = 0.9. Light transmitted through the metamaterial was imaged with a CCD camera to take polarimetry measurements (see Methods).

Beams with linear l _1,2_ = *p* = 0, ⟨σ_1_|ψ⟩ = ⟨σ_2_|ψ⟩ = 1), radial (|Rad⟩ in eq 2a), “antiradial” ( l _1_ = σ_1_ = − l _2_ = −σ_2_ = 1, *p* = 0, ⟨σ_1_|ψ⟩ = ⟨σ_2_|ψ⟩ = 1), and second-order radial ( l _1_ = − l _2_ = 2, σ_1_ = −σ_2_ = 1, *p* = 0, ⟨σ_1_|ψ⟩ = ⟨σ_2_|ψ⟩ = 1) polarization structures were investigated (Figure 2). In addition to pure radially polarized beams, which are characterized by a transverse spin, several vector beams were studied with a modified radial polarization by introducing an increasing degree of ellipticity in the transverse polarization, thus introducing also a longitudinal spin component.

**Figure 2:**
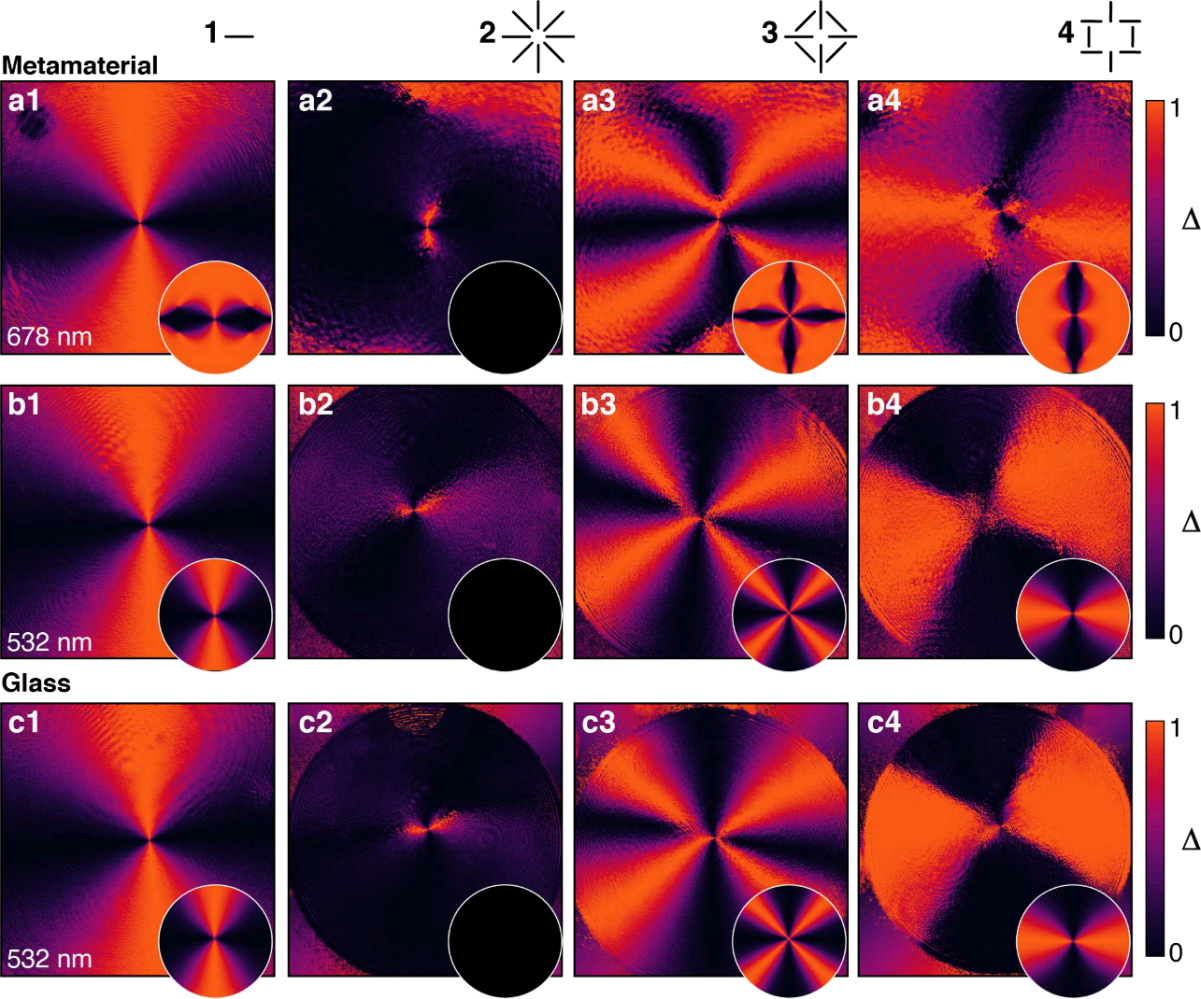
Measured projections of various states of polarization of vector beams onto the azimuthal state: (1) linear (horizontal), (2) radial, (3) “antiradial” and (4) second-order radial. The spatial maps were obtained for propagation through (a, b) the metamaterial and (c) glass for a wavelength of (a) λ_L_ = 678 nm (ENZ regime) and (b, c) λ_T_ = 532 nm (elliptic regime). Circular insets show simulation results for each case. The deviation from the azimuthal state is measured as Δ = cos^2^(ϕ_ψ_ – ϕ_Azi_).

### ENZ Regime: Polarization Azimuthalization

In the epsilon-near-zero regime (λ_ENZ_ ≈ 680 nm), the strong damping of the longitudinal field during propagation in the metamaterial causes the two-dimensional polarization of the transmitted light to rearrange into an azimuthal state (“azimuthalization”) in order to withstand the weaker longitudinal component and satisfy Gauss’s law. Azimuthalization was observed for all studied beams but the pure radial (Figure 2a), in which case the longitudinal field can never be sufficiently weakened as it is regenerated from the transverse one. The polarization is left perfectly unchanged for propagation at a wavelength far from the ENZ regime (λ_T_ ≈ 530 nm) or through the glass sample (Figure 2b,c). The changes occurring in the state of polarization were calculated from the experimental and simulated polarization distributions as a deviation of the polarization angle of the beam (ϕ_ψ_) from that of an azimuthal beam (ϕ_Azi_) as Δ = cos^2^ ϕ = cos^2^(ϕ_ψ_ – ϕ_Azi_) (Figure 1d). The experimental observations and theoretical predictions are in good agreement, with almost complete azimuthalization of the polarization of the transmitted beam.

The observed differences can be ascribed to the efficiency of the polarization conversion provided by the metamaterial, which is influenced by imperfections in the experimentally generated polarization states. Although globally reproducing the symmetry of the desired vector beams, they suffer from a remaining nonzero local ellipticity. The main consequence of this is a reduction of the longitudinal field strength, which causes a drastically lower coupling to the ENZ response of the metamaterial,^15^ diminishing the development of azimuthalization in the experiment. This can also be understood from the point of view of reduction of the transverse spin and dominating longitudinal spin. This process is restricted in pure radially polarized beams due to the requirement of zero-divergence electric field.^18^ For comparison, propagation through glass does not result in azimuthalization, as expected since glass does not influence the balance between transverse and longitudinal field components. Additionally, the local EMT, used for modeling the metamaterial transmission, might not capture all the details of the nanostructured medium,^17^ and the numerical predictions might overestimate the efficiency of the azimuthalization in the ENZ regime.

### Hyperbolic Regime: Generation of Second-Order Meron Topologies

In the hyperbolic dispersion regime, the plasmonic nanorod metamaterial offers strong anisotropy, observed at wavelengths longer than λ_ENZ_ (Figure 1c). Both strong birefringence and strong dichroism are present in this spectral range. The spin–orbit coupling enabled by elliptical polarization—and enhanced by the tight focusing—can be used to realize vortex polarization structures after transmission through the metamaterial.^14,15^ Circularly polarized beams propagating through an array of nanorods away from the ENZ regime experience a strong modification of their polarization state and vorticity (longitudinal spin and orbital angular momentum). The output polarization shows a nonuniform spatial distribution (Figure 3a,c), with the ellipticity changing with the distance from the beam center and the orientation of the local polarization creating a vortex structure whose global orientation depends on the sign of the helicity of the initial state (σ = ±1).

**Figure 3:**
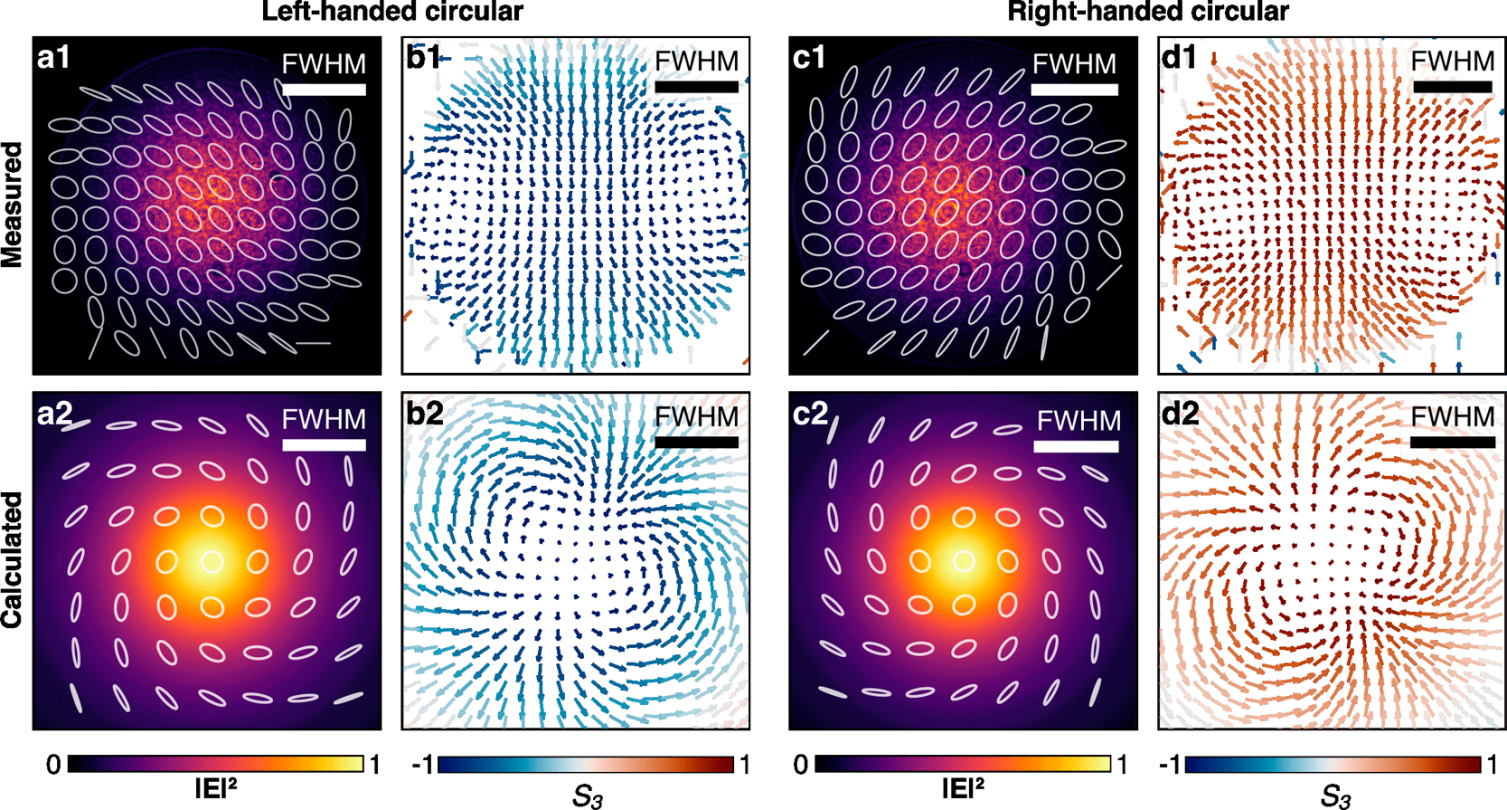
(1) Experimental and (2) theoretical results for the (left) left-handed and (right) right-handed circularly polarized Gaussian beam, tightly focused (NA = 0.85) through the metamaterial: (a, c) state of polarization recovered from the Stokes parameters (see Methods) overlapped with the intensity profile of the beam; (b, d) representation of the vector field **Σ** in the *xy* plane. The color of the arrows represents the *z* component of the field.

The origin of this state of polarization is found in the interplay between the anisotropy offered by the metamaterial and the tight focusing of the incoming circular beam. Propagating along the optical axis of a uniaxial material, a circularly polarized beam with a circularly symmetric intensity profile generates an optical vortex of order 2 with the conversion efficiency increasing with the focusing (Supplementary Figure 1). This results in the circular component of the transmitted light with opposite spin to the input,^14^ so that the angular momentum is conserved (for example, an input of σ = 1, l = 0 produces a vortex with σ = −1, l = 2 in the output). The superposition of these two components carrying different orbital angular momenta (2 and 0) and having orthogonal circular polarizations creates the state of polarization observed here. Remarkably, although the metamaterial used is considerably thin—the rod height is approximately 250 nm (≪λ)—the vortex component generated is strong enough to modify the input circular polarization. This is achieved thanks to the strong anisotropy offered by the metamaterial (Δ*n* = |*n*_*x*_ – *n*_*z*_| ≈ 1.8; see Figure 1c) and to the tight focusing (see Supplementary Figure 1), which increases the conversion rate of the input circularly polarized beam to the vortex beam of the orthogonal polarization, making it comparable to the stronger circular one and resulting in the observed polarization distribution.

The obtained structure can be described by the spatial distribution of a Cartesian vector (**Σ**) of components given by the Stokes parameters (*S*_1_, *S*_2_, *S*_3_),^10^ normalized to obtain a unit vector at every point (*x*, *y*). This reveals the emergence of a synthetic topological structure in the polarization of the beam (Figure 3b,d). The skyrmion number (SN) of this structure can be computed as^11^

3 $$SN=\frac{1}{4\pi }\iint_{\Omega }\Sigma \cdot \left(\frac{\partial \Sigma }{\partial x}\times \frac{\partial \Sigma }{\partial y}\right)\;dx\;dy$$

integrated over the *xy* plane perpendicular to the propagation direction (*ẑ*). From the numerically simulated polarization patterns, using as integration domain Ω a circle with diameter equal to the beam full width at half-maximum (FWHM), we can obtain SN ≈ ±1.05 (Supplementary Figures 2 and 3), with the sign dependent on the choice of initial helicity.

Although a unitary SN would suggest the generation of a skyrmion of order 1, the obtained topology corresponds to only partial coverage of the Poincaré sphere (see Supplementary Figures 3 and 4). The vector field **Σ** covers only one of the hemispheres, depending on the sign of the initial helicity. This observation together with the spatial distribution of **Σ** (Figure 3b,d) rather suggests that a second-order Stokes meron is observed.^21^ Conversely, there would be full coverage of the Poincaré sphere if it were a bimeron topology, which consists of two merons of opposite signs.^22^ The half-coverage shown by our results could alternatively be achieved by a meron pair, although in this case the vortex points of the two merons should be distinguishable.^23^ The topological texture in the normalized Stokes vector **Σ** can be seen as two joined merons with the same vorticity (±1/2), such that the unitary skyrmion number is explained as the sum of two half-integers with the same sign. The resulting second-order meron can be visualized in a projection of the Stokes vector field. Locally, each polarization ellipse is described by a three-dimensional Stokes vector. Projecting the polarization distribution onto the *xy* plane yields the vector field which corresponds to a second-order meron (Figure 4).

**Figure 4:**
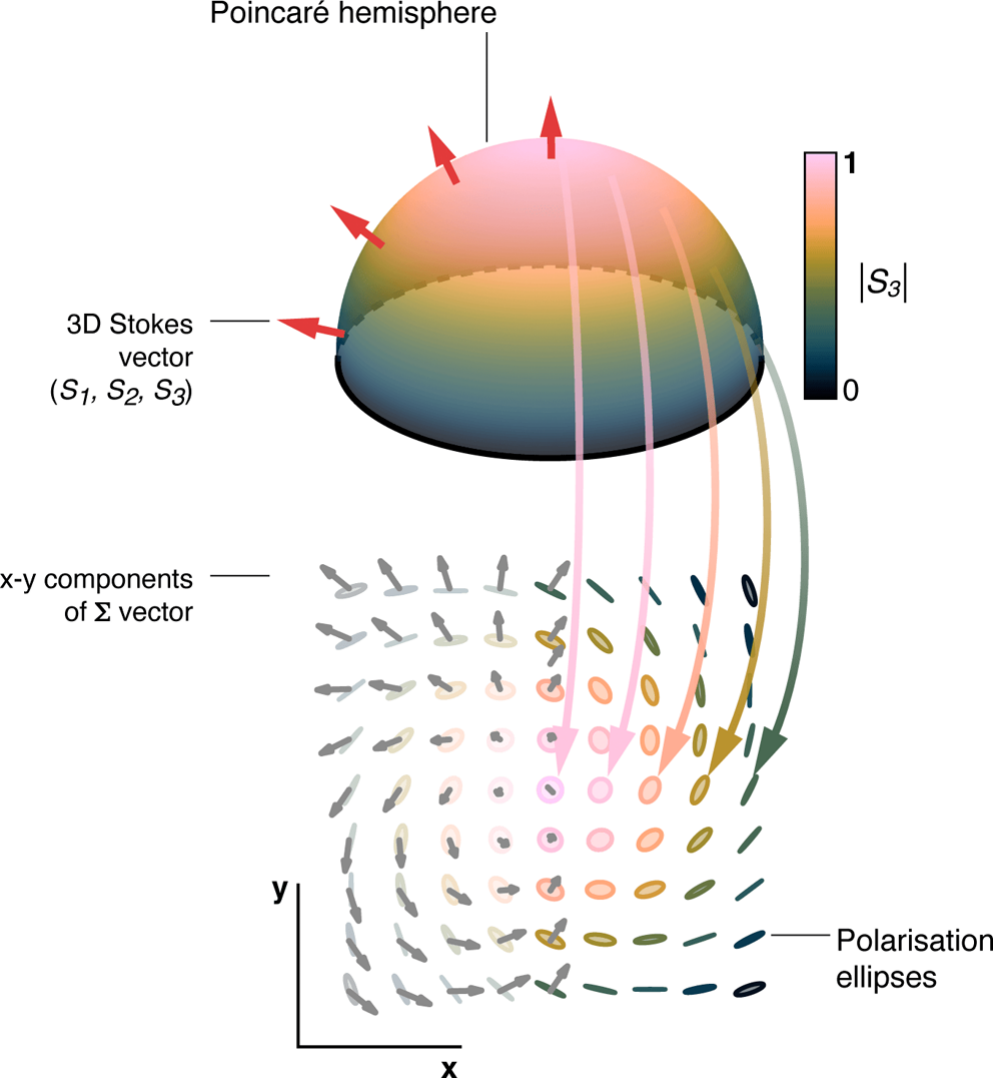
Conceptual representation of the second-order meron. (top) A hemisphere of the Poincaré sphere is shown colored according to the value of |*S*_3_| ∈ [0, 1]. A state of polarization located at any point on the sphere surface can be represented by an ellipse drawn in a plane tangential to the sphere at the same point. The vector field orthogonal to the sphere surface (shown in red) represents the Stokes vectors for each state of polarization. (bottom) The spatial distribution of the polarization states obtained from Figure 3 is shown as ellipses colored according to the value of |*S*_3_|, in relation to the hemisphere above. The gray vector field is the synthetic field **Σ**, visualized as the projection of the Stokes vector field (red arrows) onto the *xy* plane. This schematic can represent either the northern or southern hemisphere according to the specific realization of the second-order meron, provided that the directions of the vectors are changed accordingly.

Polarimetry measurements performed on tightly focused (NA = 0.85) circularly polarized Gaussian beams transmitted through the nanorod metamaterial (λ ≈ 800 nm) reproduce a state of polarization with a structure similar to that predicted by simulations (Figure 3). The experimental reconstruction of the vector field **Σ** for right (left)-handed input also shows an always positive (negative) *Σ*_*z*_, with a negligible presence of data points in the southern (northern) hemisphere of the Poincaré sphere (see Supplementary Figures 3 and 4). This results in a topological structure that does not quite reproduce the second-order meron predicted from calculations, but rather two merons of the same vorticity that are not yet joined.^21^ It should be noted that small disorder either in the metamaterial structure or the incident beam may result in the splitting of vortices leading to this observation.^24^ This results in a nonunitary skyrmion number that also fluctuates considerably with the spatial limits chosen for the integration domain Ω.

The realization of a second-order meron topology is enabled by the spin–orbit coupling, enhanced by the strong anisotropy of the metamaterial. The efficiency of this process depends on two factors: the degree of anisotropy of the metamaterial and the spin angular momentum density of the initial state of polarization. In the simulations, a gradual reduction of the ellipticity of the input beam results in the splitting of a second-order meron in two individual merons with the same vorticity. As the ellipticity decreases from circular (σ_*i*_ = ±1) to linear (σ_*i*_ = 0), the two merons drift apart and eventually vanish (Figure 5 and Supplementary Figure 5). Experimentally, the imperfections in the beam polarization primarily affect the local ellipticity, which can explain the difficulty in achieving a fully formed second-order meron even when the metamaterial anisotropy is strong enough to enable its formation.

**Figure 5:**
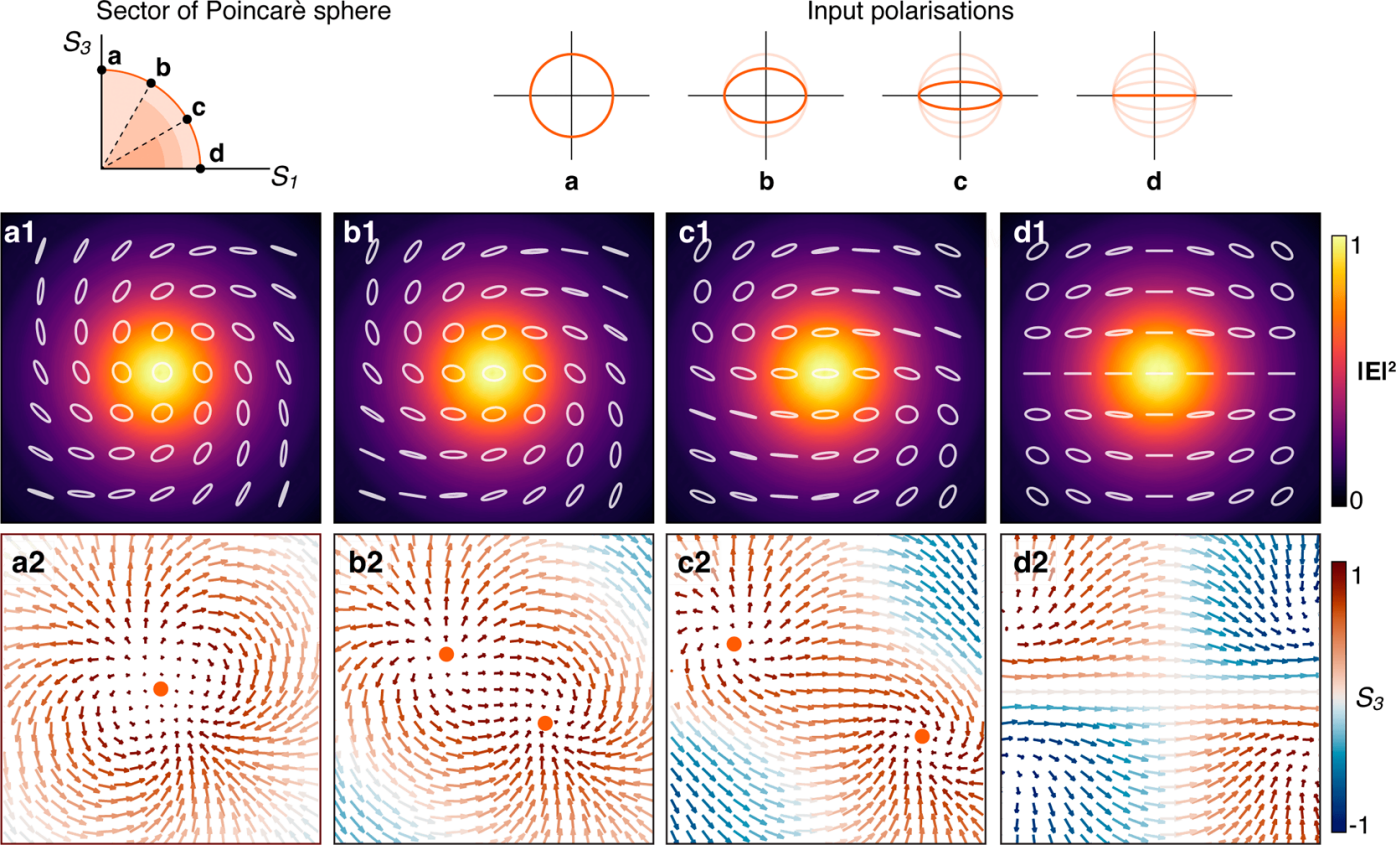
Influence of the degree of ellipticity in the initial polarization on the second-order meron topology. (a–d) The polarization changes from (a) circular to (d) linear as represented on the Poincaré sphere on the top-left inset: (1) intensity profile and state of polarization obtained after tight focusing through the metamaterial; (2) corresponding distribution of the normalized Stokes vector field **Σ**. The second-order meron in (a2) is shown to break up into single merons that drift apart as the ellipticity is reduced.

Calculations and measurements performed at wavelengths lower than λ_ENZ_ show that the anisotropy provided by the nanorod metamaterial in this range is considerably weaker than that obtained with hyperbolic dispersion. The theoretical results show an almost negligible reduction of the local ellipticity (Supplementary Figure 4) as well as the impossibility of recreating the second-order meron. The Stokes parameters obtained in this case offer considerably limited coverage of the northern hemisphere of the Poincaré sphere, which is translated into a vector field **Σ** that—although seemingly reproducing a double-meron symmetry—does not cover the full range of values needed for its *z* component, resulting in a skyrmion number of 0.17. Accordingly, the topology of interest is also lost experimentally when moving to the elliptic dispersion regime.

## Conclusions

We have studied the interaction of vector beams carrying longitudinal field with a strongly anisotropic metamaterial and the related spin–orbit coupling effects. Depending on the dispersion regime of the interaction, the metamaterial has been shown to (i) modify the beam polarization into an azimuthal state in the ENZ regime or (ii) generate a vortex-like structure in polarization with second-order meron topology—which strongly depends on the metamaterial anisotropy and the spin composition of the incident beam—in the hyperbolic regime. While the strong anisotropy offered by the hyperbolic dispersion regime leads to the realization of a second-order meron topology, the weaker anisotropy characteristic of the elliptic dispersion produces a topologically trivial texture. Experimental results consistently reproduce the theoretical predictions, taking into account imperfections in the local beam ellipticity that have been theoretically proven to drastically decrease the strength of the longitudinal field generated upon focusing.

Previously observed quasiparticles of light in the Stokes field have been generated from superpositions of optical vortices collectively possessing a nonzero total angular momentum.^10^ In this work, by tightly focusing the incident beam in a strongly anisotropic hyperbolic metamaterial, we observe the generation of Stokes merons from a simple circularly polarized beam modified by the uniaxial material. This underlines the potential of strongly anisotropic plasmonic metamaterials as a platform for beam and polarization shaping as well as controlling the topology of optical fields.

## Methods

### Metamaterial Fabrication and Characterization

The metamaterial was fabricated by an electrochemical approach as described in ref 25. The targeted parameters of the nanostructure were the radius of the individual rods *r* = 16 ± 1.8 nm, the spacing (center-to-center) between adjacent rods *s* = 60.7 ± 4.8 nm, and the overall thickness of the sample *d* = 200–250 nm.

### Semianalytical Modeling

To model the propagation of a focused beam through the anisotropic metamaterial, we have used a previously developed extension of the Richards–Wolf theory for anisotropic media.^20^ The first and last layers are considered to be free space (ε = μ = 1) and glass (ε = 2.25, μ = 1), respectively (Figure 1b). The middle layer represents the metamaterial as a bulk uniaxial crystal with the optical properties described by an effective medium theory, which models the gold nanorods as inclusions in a host alumina matrix.^17^ Using tabulated data for both materials^26^ and taking into account corrections for the quality of the electrochemical gold,^27^ the nonzero components of the effective permittivity tensor are obtained as^17^

4a $$\varepsilon_{x}=\varepsilon_{{Al}_{2}O_{3}}\frac{\left(1-f\right)\varepsilon_{{Al}_{2}O_{3}}+\left(1+f\right)\varepsilon_{Au}}{\left(1-f\right)\varepsilon_{Au}+\left(1+f\right)\varepsilon_{{Al}_{2}O_{3}}}$$

4b $$\varepsilon_{z}=\left(1-f\right)\varepsilon_{{Al}_{2}O_{3}}+f\varepsilon_{Au}$$

with

4c $$\varepsilon_{Au}=\varepsilon_{b}+\frac{i\omega_{P}\tau \left(R_{b}-R\right)}{\omega \left(\omega \tau +i\right)\left(\omega \tau R+iR_{b}\right)}$$

where *f* represents the filling fraction of the gold inclusions in the alumina matrix, the subscript b refers to quantities characterizing bulk gold, *R* is the electron mean free path for gold, ω_P_ is its plasma frequency, and τ is the average electron collision time.

### Vector Vortex Beam Generation

The setup for beam shaping (Figure 1a) is based on two reflective spatial light modulators (SLMs; HOLOEYE PLUTO-02 with a NIRO-023 head). Once the wavelength is selected from the supercontinuum source (Fianium Supercontinuum Femtopower1060 SC450-2) with a combination of filters, the beam is expanded with a pair of short converging lenses placed in a 4–*f* configuration (*f*_1_ = 35 mm, *f*_2_ = 70 mm). This allows for spatial filtering of the beam before the modulation. The reference polarization (|H⟩) is fixed by a Glan–Taylor prism (LP_1_) so that it is aligned with the horizontal axis of the SLM to maximize the modulation efficiency. The SLMs apply phase masks that encode different topological charges ( l _1_, l _2_) to the incident beam, while its polarization is rotated by a half-wave plate (HWP) to a diagonal state in between the two modulators. Using a quarter-wave plate (QWP), the copropagating vortices are made circularly polarized so that their superposition returns the desired vectorial state (eq 2). By choosing the orientation of the HWP and the QWP and the values of *l*_1_ and *l*_2_, the output polarization state can be tuned, enabling the generation of any scalar or vector beam.

### Polarimetry Measurements

A full characterization of the polarization state of the transmitted light was achieved by adding a linear polarizer and a QWP at the end of the transmission path (Figure 1a, pink area). The unknown polarization state is projected onto horizontal, vertical, diagonal, antidiagonal, and right- and left-handed circular states so as to retrieve the Stokes parameters:

5a $$S_{0}=I_{H}+I_{V}$$

5b $$S_{1}=\left(I_{H}-I_{V}\right)/S_{0}$$

5c $$S_{2}=\left(I_{D}-I_{A}\right)/S_{0}$$

5d $$S_{3}=\left(I_{L}-I_{R}\right)/S_{0}$$

The above quantities are obtained as functions of the coordinates in the transverse plane, so that the geometrical parameters of the polarization ellipse can be calculated for each pixel of the image and the local polarization can be fully characterized.

## Supplementary Material



## Data Availability

All of the data supporting the findings of this work are presented in the Results and Discussion and are available from the corresponding author upon reasonable request.

## Notes

Additional figures in support of the findings discussed herein (PDF)
