# Supplementary material for: Polarization Conversion and Optical Meron Topologies in Anisotropic Epsilon-Near-Zero Metamaterials
Source: ACS Photonics. 2025 May 15;12(6):2909–15. doi: 10.1021/acsphotonics.5c00241 (PMC12183757; doi:10.1021/acsphotonics.5c00241)
Supplement: Supplementary file 1 [file ph5c00241_si_001.pdf]

Supporting Information

Polarisation conversion and optical meron topologies in anisotropic epsilon-near-zero metamaterials

Vittorio Aita, Anastasiia Zaleska, Henry J. Putley and Anatoly V. Zayats

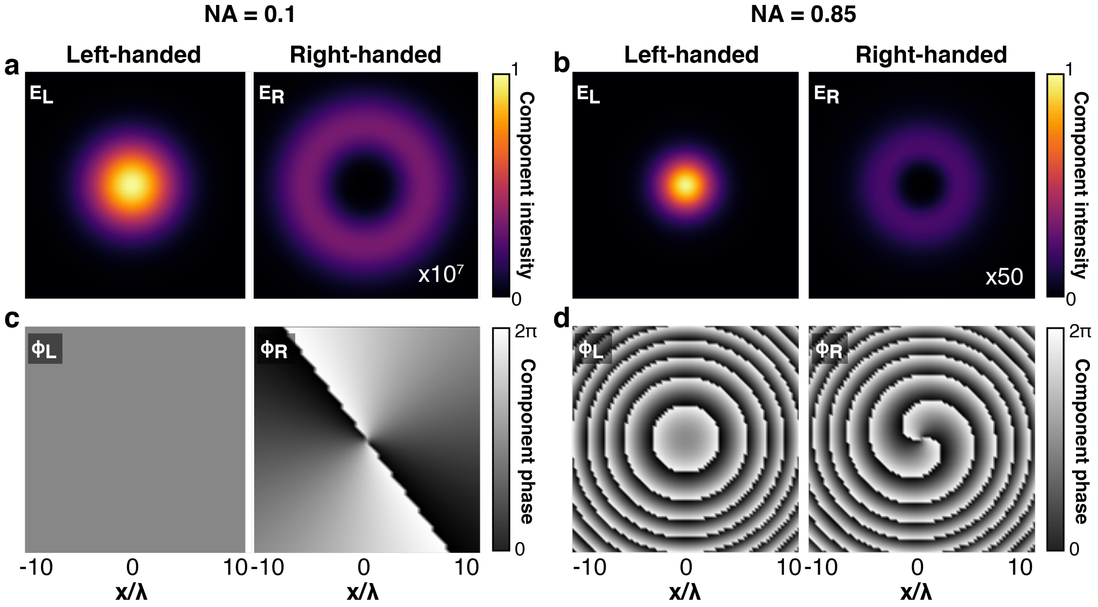

Supplementary Figure 1: Vortex generation in the metamaterial by circularly polarised light. A left-handed (L) circularly polarised beam propagates through the metamaterial at a wavelength $\lambda \approx$800 nm, red-shifted from the ENZ regime in (a,c) weak (NA = 0.1) and (b,d) strong (NA = 0.85) regime. The transmitted light is decomposed onto its (R) right-and (L) left-handed circularly polarised components as indicated by the labels in each panel. (a,b) Intensity of the R and L components as indicated by the labels. The colour scale is the same for each pair of amplitude plots, to allow direct comparison at fixed NA. The R amplitude has been scaled by a factor shown in the respective panels. (c,d) Phase distribution of the R and L components, wrapped in $[0, 2\pi]$. The effect of the tight focusing is visible as the appearance of concentric rings. The emergence of a topological charge 2 is seen as the line splitting the phase through the centre, only shown by the R components.

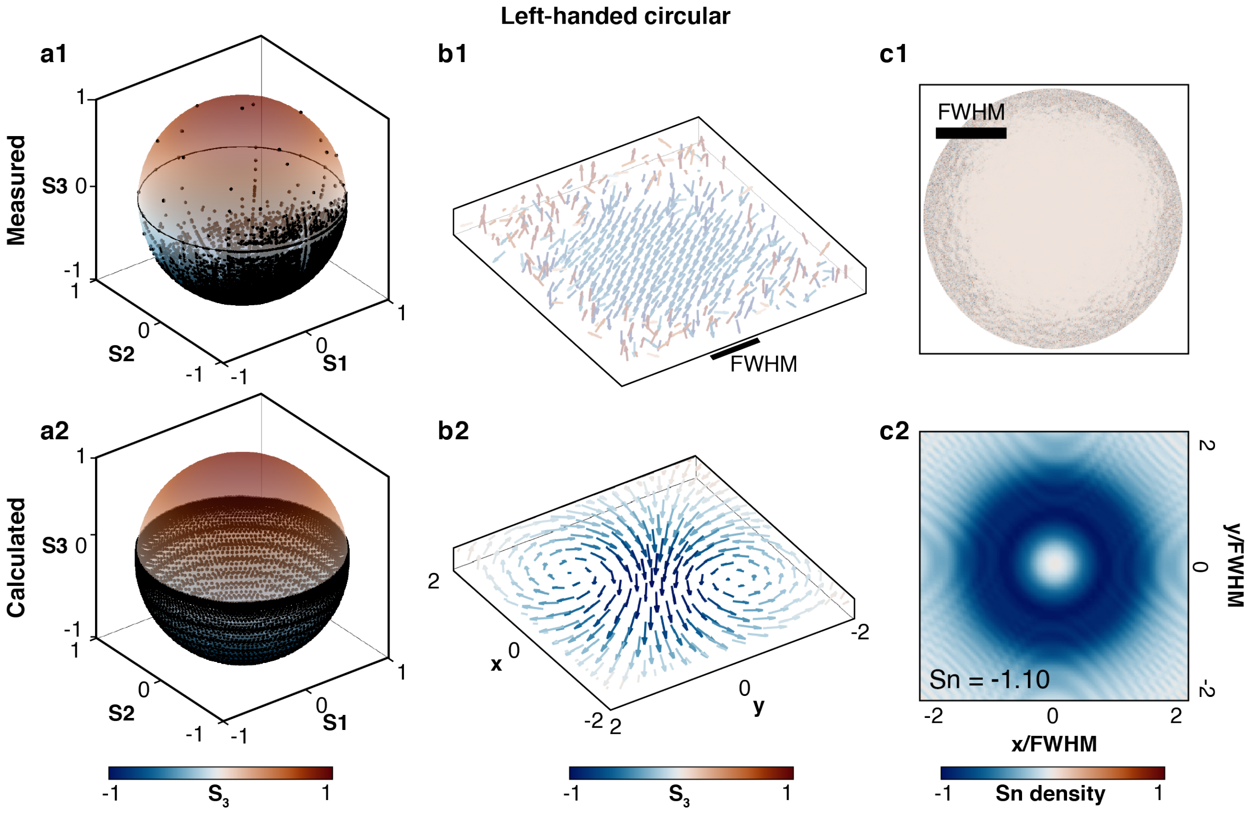

Supplementary Figure 2: Additional data for LHC. Supporting data to Fig. 3 showing (a) experimental and (b) theoretical results for a left-handed circularly polarised tightly focused Gaussian beam, propagating through the metamaterial for its hyperbolic dispersion. Analysing the state of polarisation obtained in output, the following information is retrieved: (1) coverage of the Poincaré sphere, (2) three-dimensional distribution of the vector field $\mathbf{\Sigma}$ and (3) the skyrmion number density.

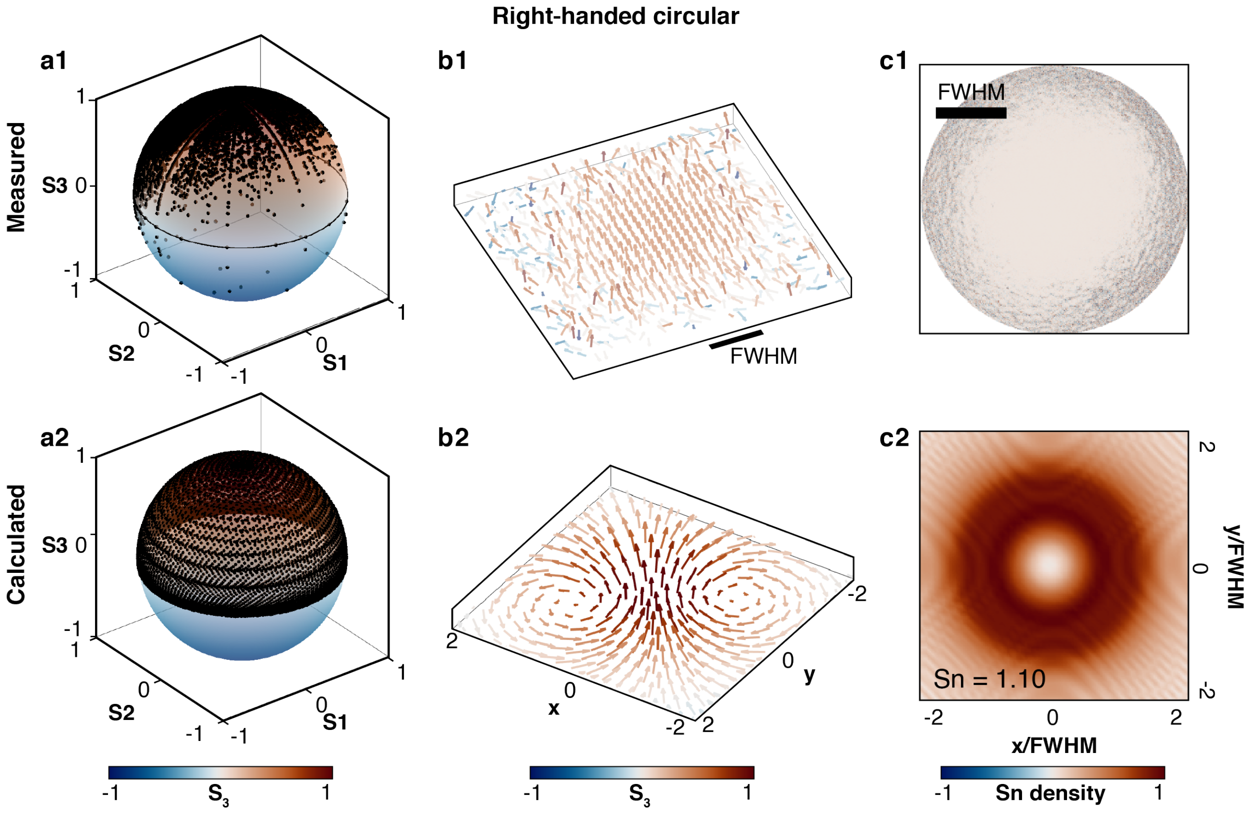

Supplementary Figure 3: Additional data for RHC. Results analogous to Supplementary Fig. 2, obtained for right-handed circular polarisation.

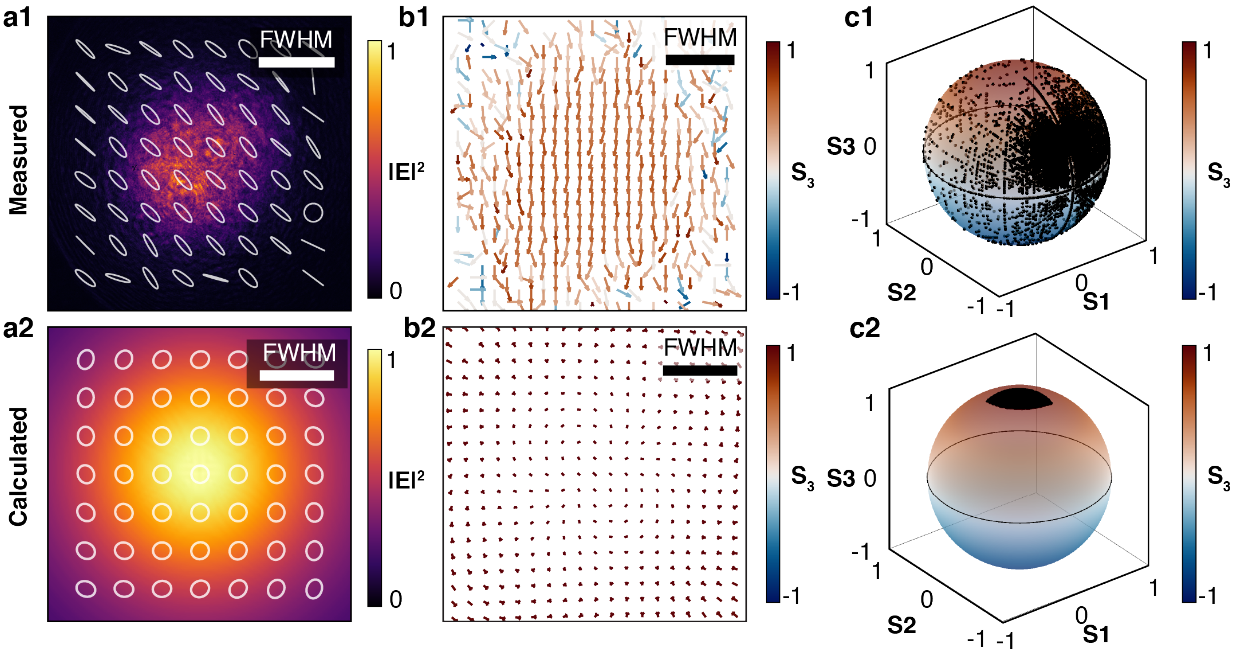

Supplementary Figure 4: Propagation in the elliptic dispersion regime. Analogous results to Fig. 3 obtained for a right-handed circularly polarised Gaussian beam and a wavelength of $\lambda = 488$ nm. (1) Experimental and (2) theoretical results for (a) the intensity profile and the state of polarisation, (b) the spatial distribution of the corresponding vector field $\mathbf{\Sigma}$ and (c) the Poincaré sphere coverage.

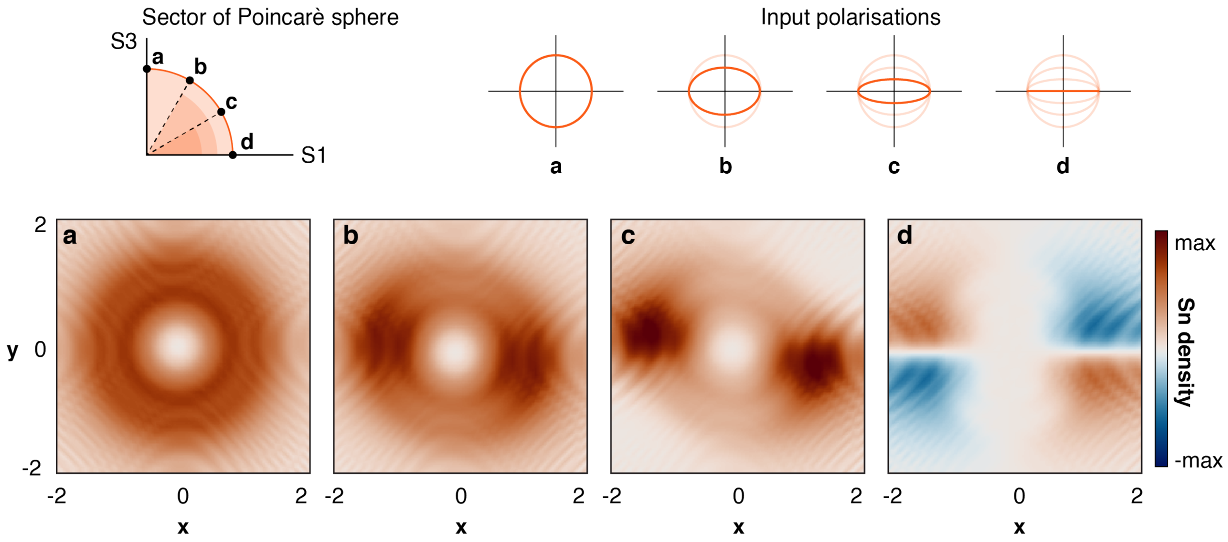

Supplementary Figure 5: Dependence of skyrmion number density on input ellipticity. Data in support of Fig. 4, showing the evolution the skyrmion number density as the input beam ellipticity is reduced from (a) circular to (d) linear polarisation.
